# Efficacy of a topical gel containing chitosan, chlorhexidine, allantoin and dexpanthenol for pain and inflammation control after third molar surgery: A randomized and placebo-controlled clinical trial

**DOI:** 10.4317/medoral.23661

**Published:** 2020-07-19

**Authors:** Luis Miguel Sáez-Alcaide, Pedro Molinero-Mourelle, José González-Serrano, Luis Rubio-Alonso, Michael M. Bornstein, Juan López-Quiles

**Affiliations:** 1 Department of Dental Clinical Specialties. Faculty of Dentistry, Complutense University, Madrid, Spain; 2 Department of Conservative Dentistry and Orofacial Prosthetics. Faculty of Dentistry, Complutense University, Madrid, Spain; 3 Applied Oral Sciences and Community Dental Care. Faculty of Dentistry, The University of Hong Kong, Hong Kong SAR, China; 4 Department of Oral Health & Medicine, University Center for Dental Medicine Basel UZB, University of Basel, Basel, Switzerland

## Abstract

**Background:**

The aim of this study was to evaluate and compare the postoperative effect of a topic gel containing chlorhexidine, chitosan, allantoine and dexpanthenol versus a placebo for pain and inflammation control after third molar surgery.

**Material and Methods:**

A gel combining 0.2% chlorhexdine, 0.5% chitosan, 5% dexpanthenol, 0.15% allantoin and 0.01% sodium saccharin was selected for this split mouth randomized controlled and double-blind trial including 36 patients with bilaterally and symmetrically impacted lower third molars. The teeth (n=72) were randomly divided into two groups before surgical removal: control group (CG; in which a placebo was given) and experimental group (EG). Swelling, trismus, postoperative pain, wound healing and complications were measured and recorded in order to evaluate differences between the placebo and experimental product.

**Results:**

Five patients suffered from an alveolitis in the CG (13.9%), and none in the study group (0%), but no statistically significant difference was found (*p*=0.063). From day 0 to day 7, trismus and swelling were significantly less pronounced in the EG, and wound healing was considered ‘good’ in 22.2% for the CG and 97.2% for the EG (*p*<0.001). Mean VAS scores during the seven postoperative days were statistically lower in the study (2.56±1,19) compared to the placebo group (3.25±1.6) (*p*=0.002). The mean consumption of analgesic pills during the first 92 hours was also statistically lower in the EG (0.26±0.51) in comparison to the CG (0.56±0.67) (*p*=0.003).

**Conclusions:**

The use of an experimental gel containing chlorhexidine, chitosan, allantoine and dexpanthenol seems to significantly reduce postoperative pain, trismus and signs of inflammation. Future studies should further evaluate, if the gel is effective in dry socket preventing after third molar removal.

** Key words:**Third molar, surgery, postoperative wound healing, pain, gel, chitosan, chlorhexidine, allantoin, dexpanthenol.

## Introduction

Third molar surgery has been described as the most common procedure in oral surgery and the postoperative phase is commonly identified unpleasant by patients (1-3). During the healing period, patients might experience pain, swelling and trismus as the most common and typical symptoms. Other complications such as alveolar osteitis (dry socket), excessive bleeding or neurosensorial alterations have also been widely described (3,4).

To reduce these symptoms and/or complications, various topical medications or gels have been suggested and investigated. Among others, chlorhexidine as an antiseptic gel or rinse has been shown to reduce these complications (4-7). Chlorhexidine has been specifically studied due to its antibacterial activity and has been demonstrated to reduce alveolitis between 24.5 to 80.2% (8). However, chlorhexidine gel application in the wound has been used by different authors without been able to find differences between chlorhexidine and placebo in terms of dry socket development and pain and swelling improvement after third molar surgery (7,8). Because of these contradictory results, chlorhexidine has been combined with other products to increase overall efficacy in more recent studies (9,10).

Chitosan is a natural polymer that comes from chitin. Its natural qualities make chitosan biodegradable, biocompatible, hemostatic, antioxidative, antibacterial, and mucoadhesive. These properties seem to be very useful in the control of postoperative complications of third molar surgery (11-13). The hemostatic benefits of chitosan in dressings are marketed for example as HemCon Dental Dressing (HDD; HemCon Medical Technologies, Inc, Beaverton, OR). The control of postoperative hemostasis following oral surgery procedures using this product has already been described (14). On the other hand, it is known that chitosan can be useful in reducing postoperative pain and inflammation, as well as decreasing bacterial proliferation after periodontal and peri-implant disease treatment (12). In addition, recent pre-clinical studies suggest that chitosan gel generates a protective hydrolipidic layer that prevents and improves tissue perfusion, thus promoting tissue regeneration and providing anti-inflammatory characteristics (13).

Recently, a topical gel that contains 0.2% chlorhexidine, chitosan, allantoin and dexpanthenol has been commercialized. Since it is known that all these components have antiseptic, anti-inflammatory and tissue repairing properties (12), it has been suggested that this topical gel could have beneficial effects on postoperative healing after third molar surgery. To our knowledge, no previous studies comparing this product versus placebo in the postoperative period after surgical removal of lower third molars have been performed.

The aim of this study is to evaluate the effect of a topical gel that contains 0.2% chlorhexidine, chitosan, allantoin and dexpanthenol compared to placebo in terms of pain, swelling and wound healing after third molar surgery. The null hypothesis was that there is no difference between the topical gel (test) and placebo (control) for postoperative outcomes (pain, swelling, trismus, wound healing) after lower third molar surgery.

## Material and Methods

This randomized and controlled clinical trial was designed following CONSORT guidelines (15). The protocol was evaluated and approved by the Research Ethics Committee at the Clínico San Carlos Hospital of Madrid, Spain (Trial registration code CEIC 17/386-R_X). Informed consent was obtained from all participants in writing prior to conducting the research.

- Patient selection

The patients were selected from those attending the Postgraduate Clinic in Oral Surgery and Implant Dentistry, Faculty of Dentistry, at Complutense University of Madrid between January 2018 and May 2019 until the required sample was obtained. The screening examination included the following: medical and dental questionnaire, standardized panoramic radiograph made at the Dental Radiology Service, Faculty of Dentistry, Complutense University of Madrid (CS 9300®, Carestream Dental, Atlanta, GA, USA), and signed informed consent.

To be included in the study the subjects had to fulfill the following inclusion criteria: aged 18 years or older, patients ASA 1 according to the American Society of Anesthesiologists classification (16), indication of lower third molar surgery, similar surgical difficulty on both sides according to Pederson scale (17), no known allergies to any of the gel components (chitosan, clorhexidine, allantoin, dexpanthenol), the medications prescribed (amoxicillin, ibuprofen, dipyrone) and the anesthetic solution used (4% articaine with 1:100,000 adrenaline).

Exclusion criteria were as follows: patients who did not agree to participate in the study, patients who did not understand the procedures of the study, patients who did not come at 48 hours and at 7 days follow-up, smokers (more than 10 cigarettes per day), patients treated with antibiotics or anti-inflammatory medication 4 days prior to the surgery, pregnant or lactating women and healthy periodontally patients with no residual pockets > 4 mm.

- Sample size calculation

The sample size was calculated similarly to the study by Haraji *et al*. (18) that used a 0.2% clorhexidine bioadhesive gel in comparison to a placebo for the prevention of alveolar osteitis after mandibular third molar surgery. For the present study, it was estimated that less than 5% of the study group would suffer from an alveolitis. Thus, it was estimated that 32 patients in each group would be required to obtain 90% power to detect this effect as statistically significant (=0.05). As a 15% loss to follow-up was assumed, the number of subjects per group to reach the objectives was indicated as 74, i.e. 37 per group since this investigation is conceived as a split-mouth study.

- Study design

To assess the differences between the placebo and the experimental product, a split-mouth randomized placebo-controlled trial was conducted. Once included, lower third molars interventions of the patients were randomly divided through a digital platform (Viedoc©, Pharma Consulting Group, Uppsala, Sweden). Prior to the surgical procedure, one blinded examiner (P.M.M.) recorded the baseline data, including the following: reason for the surgery, interincisal distance measured in millimeters to evaluate trismus, and facial perimeter to analyze postoperative swelling according to Amin and Laskin (19), which measures the changes in millimeters before and after surgery between different facial points including the distance from gonion to the external canthus of the eye (Go-Eye), the distance from tragus to the labial commissure (Tg-Com), and the distance from tragus to pogonion (Tg-Pg).

- Surgical procedure

All surgical procedures were conducted by a single surgeon (L.M.S.A.) between 8:00 am and 11:00 am. A minimum of one month was allowed between one surgery and the intervention on the contralateral side. The anesthetic used for all interventions was 4% articaine with 1:100,000 adrenaline (Laboratorios Normon; Tres Cantos, Madrid, Spain).

An envelope-shape incision from the lower second molar with a vertical releasing incision on the ramus was made, and a mucoperiosteal flap was raised to expose the lower third molar. No. 8 tungsten carbide bur on a surgical handpiece was used to perform bone removal and, if necessary, to section the molar. After the tooth extraction, bony edges were smoothened, and the wound was irrigated with copious use of saline solution. Then, the flap was sutured with simple interrupted sutures using 4.0 silk (Laboratorio Aragó S.A.; Barcelona, Spain).

According to the previously prepared randomization, experimental gel or placebo were applied on the wound (14). Finally, the surgeon filled out the data collection sheet using the vial code for further processing. Surgery time, surgery difficulty according to Parant scale (18), and surgical complications were also recorded.

Patients were prescribed amoxicillin 750 mg to be taken every 8 hours for 7 days, and Ibuprofen 600 mg every 8 hours for 4 days. They were also prescribed 575 mg of dipyrone for use as a rescue analgesic as need.

- Follow-up

During the postoperative week, patients in the experimental group applied topical gel composed of chitosan, 0.2% chlorhexidine, allantoin and dexpanthenol (Bexident® Post Tratamiento Gel Tópico, ISDIN S.A.; Barcelona, Spain) on the surgical wound.

Patients in the control group applied the placebo gel, which was manufactured from a master pharmacological formulation including all the components from the commercially available gel with the exception of chitosan, 0.2% chlorhexidine, allantoin and dexpanthenol. Patients in both groups applied the gel three times a day for 7 days.

During the follow-up periods at 48 hours and 7 days the examiner (P.M.M.) recorded the following data: facial perimeter measures and interincisal distance to evaluate changes compared to baseline, the wound healing using a modified scale according to Madrazo-Jimenez *et al*. (14) (Table 1), presence of alveolar osteitis according to the Blum criteria (20), and infectious, hemorrhagic or neurosensorial complications. Postoperative pain was measured using a visual analog scale (VAS) from 0 to 10, where 0 supposes no pain and 10 the worst possible pain, during the first seven postoperative days. Furthermore, the number of rescue analgesic taken during the first four days after surgery were recorded.

**Table 1:**
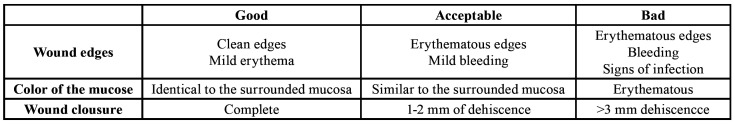
Scale used to evaluate wound healing.

- Statistical analysis

Descriptive parameters (frequency, mean and standard deviation) of the variables were calculated. For the quantitative variables (facial perimeter, trismus, postoperative pain), the Student's T test for paired samples and the Wilcoxon signed-rank test were used. For the categorical (ordinal) variables (surgery difficulty, presence of alveolar osteitis and wound healing), the McNemar and Wilcoxon tests were performed (*p*<0.05).

The significance level chosen for all statistical tests was *p*<0.05. Statistical analyses were all performed using the IBM SPSS Statistics v.25 software (IBM, United States).

## Results

**Figure 1:**
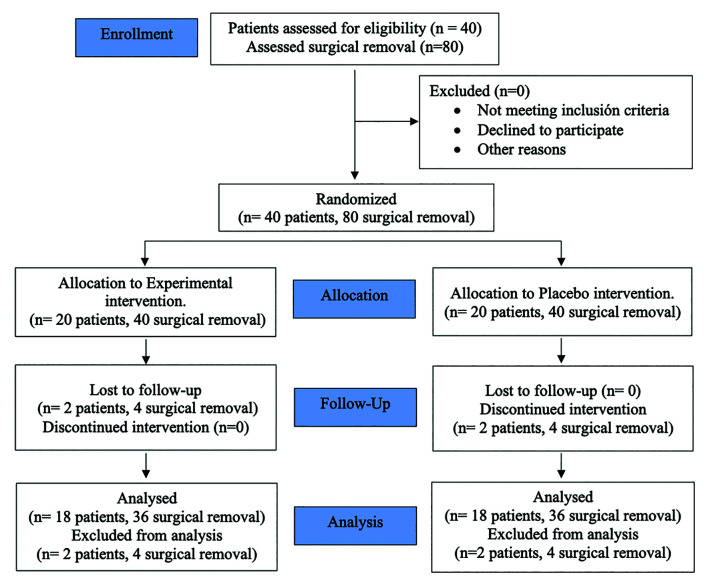
Flowchart diagram of the study protocol according to Consort guidelines.

Forty patients were recruited to participate in this study. Thirty-six patients completed the study (23 women and 13 men; mean age 22.94±2.67 years). Two patients of the placebo group did not want to perform the second left or right third molar surgery, and two patients did not come to the follow-up visits in the study group. Therefore, these patients were excluded from further analyses (Fig. 1). Age and gender were the same for both groups, since a split-mouth design was performed. The surgical difficulty of the third molars and the time for the surgical interventions are shown in Table 2. In this study, the duration of the surgeries did not exceed 30 minutes for any of the cases included.

**Table 2:**
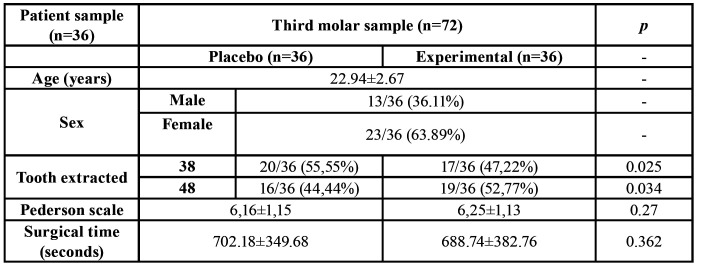
Characteristics of patients and surgical variables analysed.

- Clinical outcomes

Alveolitis

The presence of an alveolitis was reported in 5 out of 36 sockets (13.9%) in the placebo, and none (0%) was recorded for the study group (*p*=0.063).

Swelling

Mean changes for facial swelling from baseline to the visit at 48 hours and 7 days after surgery are presented in Table 3. Statistically, lower differences were observed for Tg-Pg values in the study group compared to the control group between baseline and the 48 hours visit (*p*=0.023). Trismus and all three swelling measurements showed significantly lower values in the study group compared to the control patients from day 0 to day 7 (Go-Eye, *p*=0.009; Tg-Com, *p*<0.001; and Tg-Pg, *p*<0.001).

Wound healing

**Figure 2:**
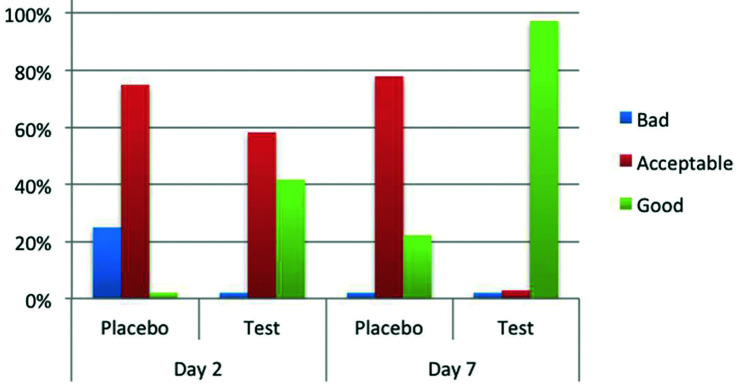
Wound healing during the postoperative period.

Two days after surgical removal of the lower third molars, wound healing was considered “bad” in 25% and 0%, “accepTable” in 75% and 58.3%, and “good” in 0% and 41.7% of the sockets in the placebo and study groups (*p*<0.001), respectively (Fig. 2). Seven days after surgery, wound healing was considered “bad” in 0% and 0%, “accepTable” in 77.8% and 2.8%, and “good” in 22.2% and 97.2% of the cases for the placebo and study groups (*p*<0.001), respectively (Fig. 2).

**Table 3:**
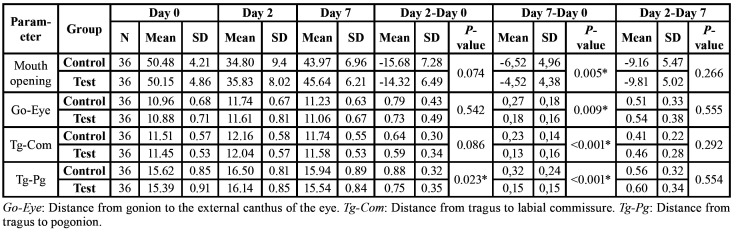
Mean and standard deviations (SD) of mouth opening and facial perimeter in test and placebo groups during the postoperative period.

Pain

**Figure 3:**
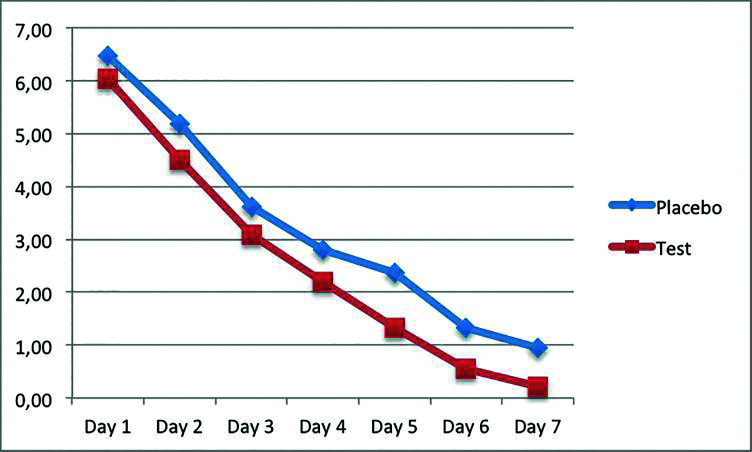
VAS scores from day 0 to day 7 in each group.

The mean VAS values for the 7 days following surgical removal were 3.25±1.6 and 2.56±1.19 in the placebo and study groups (*p*=0.002), respectively (Fig. 3). A mean consumption of 0.56±0.67 and 0.26±0.51 analgesic pills per day was recorded for the placebo and study groups during the first four days after the surgeries, respectively (*p*=0.003).

- Complications and adverse effects

For the test group, one case of hematoma was reported. In the placebo group, 3 patients with hematoma and cases and finally 1 case with temporary paraesthesia of the inferior alveolar nerve were seen.

## Discussion

The aim of this study was to evaluate the efficacy of a topical gel containing 0,2 % chlorhexidine, chitosan, allantoine and dexpanthenol compared to a placebo in the postoperative healing period after lower third molar surgery. To the best of our knowledge, this is the first study published that compares this product versus a placebo. However, there are some limitations of the present study that should be considered for further studies on this topic. The assessment of the clinical outcomes, especially pain and wound healing, has been done using subjective scales, which could be conceived as biased. Moreover, further studies should focus on analyzing the role of the gel tested on postoperative swelling and should also include a longer follow-up period.

Moreover, a longer follow-up period would be better in order to analyze in more detail the evolution of the clinical results, especially the development of alveolitis (6).

It has been reported in the literature that time is relevant in oral surgery, and that there is a direct relationship between the increase in postoperative inflammation and pain and a prolonged duration of the procedures. Therefore, this will also impact on the subsequent healing process (3). In the present study, the longest surgery lasted for 25.30 minutes, which is not considered over the limit of 30 minutes (3,21).

The most striking differences between the two groups analyzed were found in wound healing and postoperative pain with better results for the experimental group. Although there were no significant differences between groups with regard to alveolar osteitis, our results suggest that the experimental product could be effective in alveolar osteitis prevention as there have been no cases of alveolitis in the experimental group.

The antiseptic effect of topic chlorhexidine in the oral cavity is well documented and improved wound healing and a preventive effect with regard to alveolar osteitis has been demonstrated after topic application of chlorhexidine gel or rinses following third molar surgery (22,23). Chitosan has also antiseptic action due to its antimicrobial power against gram-negative, gram-positive bacteria and fungi. In recent years, tissue repairing properties have also been documented for chitosan, which could be promising for periodontal/guided bone regeneration (24). Furthermore, dexpanthenol has been widely studied in the field of dermatology, where *in vivo* and *in vitro* studies have demonstrated a positive effect on the proliferation of human fibroblast, which could result in improved soft tissue healing (25). All these favorable findings of antiseptic and tissue repair capacities attributed to chlorhexidine, chitosan and dexpanthenol could be directly related to an improvement in wound healing after third molar surgery, which could explain the results seen in the present study.

However, despite the positive effect of these components, it is not possible to assume that the topical application of the gel tested would have enough power to result in any evident systemic effects.

At the second day, 25% of the cases (9 patients) in the placebo group showed “bad” (unsatisfactory) healing compared to the experimental group that showed only "accepTable" or "good" wound healing. All the previous mentioned properties of the experimental gel could explain these differences. Nevertheless, the method chosen here to evaluate wound healing could be rather subjective, and therefore, the results should be interpreted with some caution.

As for bad (unsatisfactory) wound healing, alveolar osteitis was observed only in the placebo group (19,3%), but not in the experimental group. Although significant differences were not found between groups, the preventive effect of chlorhexidine in terms of dry socket and the antiseptic effect of chitosan could explain these results. Moreover, the 5 patients who developed alveolar osteitis in the control group were in need of extended surgical procedures including bone more removal and tooth sectioning due to the deeper position of the third molars. Here, the mean surgical time was 20:34 minutes. These factors could also at least partially explain the differences seen between the two groups.

Regarding other complications, a transitory paresthesia of the inferior alveolar nerve was observed in the control group. The patient presented a very deep impacted third molar with a direct relation to the inferior alveolar nerve. Extended bone removal including crown and root sectioning were needed to remove the tooth. This intervention lasted for 22:43 minutes from incision to suturing. This could very likely explain the complication seen. For the paresthesia, no treatment was needed, and total recovery was recorded 14 days after surgery. Additionally, 4 cases of hematoma were seen, two of them in the same patient. In all these cases, the surgeries included bone removal and tooth sectioning were needed, and the interventions lasted 15:32 minutes (mean). Thus, the complications mentioned here do not seem to have a direct relation with the application of either placebo or experimental product, but rather with difficulty of the surgery itself and the surgical time needed.

Similar results to ours have been published in the study of Madrazo-Jimenez *et al*. (14). In this research they applied a gel containing chitosan in the experimental group but did not use a placebo in the control group. As our results, the authors found a statistically significant difference for wound healing in favour of the experimental group. However, they did not find differences in terms of pain between the groups. One reason that may explain this discrepancy is the subjectivity and individual variability of pain assessment using VAS that can result in different findings especially for studies with limited power.

López-López *et al*. also evaluated the effect of this gel following third molar surgery in a randomized controlled study comparing the efficacy of this product compared to a bicarbonate oral rinse (13). In terms of pain, they found better results in favor of the gel containing chitosan with significant differences for VAS values and analgesic intake. Likewise, better wound healing was recorded for the experimental group. These clinical findings were very similar to the ones recorded in the present study. Based on these results, it seems possible that this product could be effective in reducing postoperative pain and improving wound healing after surgical removal of third molars.

Several products have been used to improve healing and reduce complications after surgical third molar extraction. Chlorhexidine is probably the most studied antiseptic in this sense. Rodríguez-Sánchez *et al*. carried out a systematic review and they concluded that both chlorhexidine rinse and gel application was effective to prevent alveolar osteitis after third molar extraction (22). Similar results were found in another systematic review published one year later, in which it was concluded that chlorhexidine gel application was superior to placebo in reducing the incidence of alveolitis after mandibular third molar surgery (23). Despite all these positive properties, several side effects such as discoloration of teeth and oral mucosa, a burning sensation or taste disturbances have been attributed to chlorhexidine (26). These aspects should be considered when prescribing it after third molar surgery. In view that the gel tested does not only contain chlorhexidine but also other components which improve clinical outcomes, such a more wholistic formulation could be more complete and effective to prevent and reduce complications after third molar surgery including impaired wound healing or postoperative pain.

Povidone-iodine or Betadine is another of the most widely used antiseptic agents in surgery. Hasheminia *et al*. conducted a clinical study with 189 patients in which they compared the efficacy of 1% betadine solution rinses versus a control group without any antiseptic. The results showed a significant difference between groups in favor of betadine rinses for the incidence of dry socket formation after third molar surgery. However, there is no evidence that betadine also improves wound healing and decreases postoperative pain (27). Therefore, the experimental gel used here could be more effective than betadine after third molar surgery.

Hialuronic acid has also been widely applied in daily practice after third molar surgery. Koray *et al*. evaluated the efficacy of hyaluronic acid spray versus benzydamine hydrochloride in reducing swelling, pain and trismus after mandibular third molar surgery. Although they found no evidence of a reduction in pain levels between groups, hyaluronic acid offered clinical benefits in the management of swelling and trismus for the immediate postoperative period. In their study, none of the patients develop an alveolar osteitis in any of the groups. These findings may be directly related to repairing tissue properties of hyaluronic acid, which could improve wound healing and, in consequence, reducing inflammation (28). Although the positive properties of hyaluronic acid on tissue repair are well documented, the tested experimental product seems to be more effective after third molar surgery by not only improving wound healing but also preventing alveolar osteitis.

Propolis is a natural product substance obtained from beehives, and it has been attributed with anti-bacterial, anti-fungal and anti-inflammatory properties. In the field of oral surgery, it has been demonstrated to have positive effects in the treatment of oral aphthous ulcers and in the prevention of bacterial colonization on sutures after oral surgery (29,30). To the best of our knowledge, there are no studies that have evaluated its effectiveness after third molar surgery. Therefore, further studies on this topic are needed, and a gel containing propolis cannot be an alternative at the moment to the gel tested here.

## Conclusions

Based on the findings of the present study, the tested topical gel containing 0.2% chlorhexidine, chitosan, allantoin and dexpanthenol results in a significant reduction of postoperative pain, trismus and inflammation compared to the placebo used. Furthermore, improved wound healing capacities can be seen in the experimental group.
